# vivoBodySeg: Machine learning-based analysis of C. elegans immobilized in vivoChip for automated developmental toxicity testing

**DOI:** 10.21203/rs.3.rs-4796642/v1

**Published:** 2024-09-04

**Authors:** Andrew DuPlissis, Abhishri Medewar, Evan Hegarty, Adam Laing, Amber Shen, Sebastian Gomez, Sudip Mondal, Adela Ben-Yakar

**Affiliations:** vivoVerse, LLC; vivoVerse, LLC; vivoVerse, LLC; vivoVerse, LLC; vivoVerse, LLC; vivoVerse, LLC; vivoVerse, LLC; vivoVerse, LLC

**Keywords:** U-Net, few-shot learning, C. elegans, developmental toxicity, microfluidics, high-throughput screening

## Abstract

Developmental toxicity (DevTox) tests evaluate the adverse effects of chemical exposures on an organism’s development. While large animal tests are currently heavily relied on, the development of new approach methodologies (NAMs) is encouraging industries and regulatory agencies to evaluate these novel assays. Several practical advantages have made *C. elegansa* useful model for rapid toxicity testing and studying developmental biology. Although the potential to study DevTox is promising, current low-resolution and labor-intensive methodologies prohibit the use of *C. elegans* for sub-lethal DevTox studies at high throughputs. With the recent availability of a large-scale microfluidic device, vivoChip, we can now rapidly collect 3D high-resolution images of ~ 1,000 *C. elegans* from 24 different populations. In this paper, we demonstrate DevTox studies using a 2.5D U-Net architecture (vivoBodySeg) that can precisely segment *C. elegans* in images obtained from vivoChip devices, achieving an average Dice score of 97.80. The fully automated platform can analyze 36 GB data from each device to phenotype multiple body parameters within 35 min on a desktop PC at speeds ~ 140x faster than the manual analysis. Highly reproducible DevTox parameters (4–8% CV) and additional autofluorescence-based phenotypes allow us to assess the toxicity of chemicals with high statistical power.

## Introduction

Traditional developmental toxicity (DevTox) studies have relied on mammalian models such as mice, rats, and rabbits to study adverse effects on their development when exposed to chemicals. Scientific and technological advances have led to the development of new approach methodologies (NAMs), such as *in silico, in vitro,* or small model organisms (*C. elegans, daphnia,* zebrafish embryo), reducing the use of vertebrates. Among model organisms, *C. elegans,* has several unique advantages, including small body size, ease of culture, homologous genes to humans, conserved xenobiotic pathways, several organ systems, etc., making it suitable for high-throughput toxicology screening platforms. Developmental parameters from *C. elegans* models have demonstrated high concordance with mammalian toxicity endpoints and outperformed other small model organism species in some situations [1–9].

Although *C. elegans* have been used as a model organism in major discoveries in the fields of neurodegeneration, aging, and toxicology assessments [5, 9–18], these studies either had low throughput or used gross phenotypes. Specifically, the automated studies were performed by either taking low-resolution images of *C. elegans* from well plates [19–22] or 1D scattering signal using a flow cytometer to extract developmental parameters [2, 23–25]. Both technologies rely on using anesthetics to reduce worm movement, which is known to have adverse effects on worms, causing their bodies to shrink or curl, eventually introducing errors in the body length measurements [22, 25]. Flow cytometers can provide such data from a large number of worms, however, with high variability (coefficient of variance, CV > 20%) [26]. Plate-based assays also demonstrate poor statistical power due to the small number of worms used per well to avoid overlap and thus simplify image analysis [22, 26–28].

We previously developed a microfluidic-based immobilization method to collect high-resolution images of many *C elegans* without using anesthetics and eliminate their random orientation [29–32]. This microfluidic device, called vivoChip, facilitates rapid immobilization of thousands of *C. elegans* from multiple different populations in parallel microchannels. Specifically, the vivoChip enables collecting high-resolution images from 40 microfluidic channels for each population of *C. elegans* with each worm body spanning over thousands of pixels (5,056 × 354 pixels per channel). Imaging a long microchannel allows us to capture the entire *C. elegans* body in a single field of view (FOV) and avoid image stitching. The vivoChip can immobilize worms with a wide range of body sizes, which is an outcome of the adverse effects on worms’ development and health when exposed to high concentrations of toxic chemicals. Such images of worms with different body sizes demonstrate a variety of contrast levels, resulting in additional challenges during image analysis.

DevTox analysis of such high-resolution data obtained from vivoChip devices is very time-consuming and necessitates automation. Several machine learning (ML)-based vision systems have been developed for the segmentation of *C. elegans* bodies in images obtained by plate readers to analyze their growth and behavioral parameters [33, 34]. These systems, however, face difficulties in multi-object identification in a dense setting with overlapping specimens [35]. Imaging *C. elegans* using vivoChip overcomes this concern by immobilizing up to 40 worms side by side within 40 parallel microchannels per population. Automated *C. elegans* body segmentation of the vivoChip images requires processing full 3D image stacks to precisely detect the worm and correctly identify its boundaries from high-frequency content that spans the entire volume.

In this paper, we present vivoBodySeg, an ML-based model to automatically segment *C. elegans* bodies immobilized inside the vivoChip devices and, thus, streamline accurate and multiparametric DevTox studies. To create extensive and balanced ground truth data, we developed a user-friendly toolbox that enables the visualization, classification, and segmentation of vivoChip-generated *C. elegans* images. vivoBodySeg utilizes a 2.5D U-Net architecture with an attention mechanism at the bottleneck that is trained for classification and semantic segmentation of the *C. elegans* body. The model achieves highly accurate segmentation with a Dice score of 97.8% across a heterogeneous population of *C. elegans*. The predicted segmentations are indistinguishable from humans while taking ~ 150x less time. Further, we demonstrate that with a careful fine-tuning procedure using only a small number of samples and four hours of training, we are able to segment a phenotypically disparate population with a Dice score of ~ 97%, providing a 2% improvement to the based model. This automated image analysis pipeline reduces human error, eliminates user bias, and achieves repeatable high-accuracy analysis of DevTox parameters.

## Methods

### C. elegans culture and chemical treatment.

*C. elegans* culture and chemical treatment are performed according to previously published protocols [29, 30, 36]. Briefly, N2 *C. elegans* strains (*Caenorhabditis* Natural Diversity Resource - CaeNDR) are collected from gravid adults by sodium hypochlorite treatment and developed into synchronized larvae 1 (L1) stage worms overnight. L1s are placed in a 24-well plate with HB101 food in S media. The L1 larvae are treated with Methylmercury (II) hydroxide (CH_3_Hg, CAS# 1184-57-2, Sigma), a known developmental toxicant [37, 38], in conventional 24-well plastic plates with solvent controls. The plates are incubated at 20°C for 72 hours until the worms in the control wells reach the day 1 (D1) stage of adulthood. The experiments are repeated five times using five vivoChip-24x devices on three different days.

### High-resolution imaging of C. elegans.

**Figure 1:**
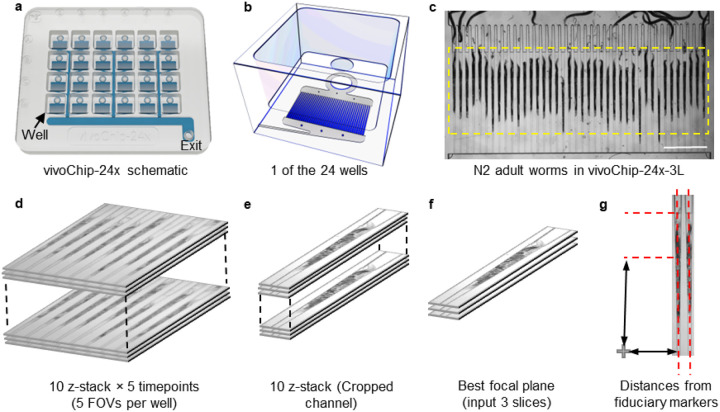
High-resolution *C. elegans* body images are acquired using a vivoChip-24x platform. (**a**) Schematic of vivoChip-24x technology to immobilize *C. elegans* and capture their high-resolution images from 24 different populations and 40 worms per population. (**b**) Schematic of 1 well out of the 24 wells. The top of the device has a well. Underneath each well, there are 40 parallel immobilization channels. (**c**) Brightfield image of 40 adult *C. elegans* immobilized inside microfluidic channels within vivoChip-24x-3L device. Scale bar is 1 mm. (**d**) Graphic to demonstrate a z-stack of 10 images collected for each FOV (8 channels per FOV with 10x, 0.4 NA objective). For each FOV, we collect 50 images over 5 time points and 10 z-stacks (z-step size of 6 μm) per time point (1 s time interval). (**e**) We crop each channel for the first time point. (f) We identify the best focal plane for analysis and use 3 slices, including the image below and above the focal plane to train the network. (**g**) The location of the worm within the channel is used to determine the channel height based on its position with respect to the fiduciary cross mark present within each well of the vivoChip-24x.

To acquire high-resolution images of worms, we load all 24 populations into a 24-well microfluidic device (vivoChip-24x, vivoVerse) in M9 buffer after 72 hours of chemical treatment in conventional plates. Underneath each well of the vivoChip-24x device, there are 40 parallel, gently tapering 3 mm long microfluidic trapping channels to trap *C. elegans* (Figs. 1a–c). The vivoChip-24x device contains a total of 960 trapping channels. A custom-designed gasket seals the device to provide fluidic connections to all wells. A single input in the gasket applies fluidic pressure to push the worms into individual channels (1 animal per channel) using intermittent ON/OFF fluidic pressure cycles. Once all channels fill up with worms as they are immobilized inside the parallel, narrowing channels, a constant fluid pressure holds them still for performing blur-free imaging. Automated high-resolution imaging is then performed on all 960 channels to collect time-lapse and z-stack brightfield images, and z-stack fluorescence images within 30 minutes using a customized automated microscope (IX73, Evident) with a high-quantum efficiency, fast, and large area camera (IRIS15, Teledyne). All 40 channels underneath each well are imaged in 5 FOVs using 10x, 0.4NA objective. Each FOV includes 8 channels. The entire worm volume is captured with 10 z-slices at 6-micron steps centered around the best focal plane of a fiduciary marker. We also collected 5 time-lapse 3D hyperstack images at 1-second intervals (Fig. 1d). Following the time-lapse brightfield imaging, a single z-stack of autofluorescence images is also acquired using a GFP filter set using the same objective.

There are 2 types of vivoChip-24x devices used in this study to accommodate the complete immobilization of *C. elegans* of different body sizes. The first device, the vivoChip-24x-3L device, has 3-layer microchannels with different heights that can immobilize young adult (YA) to Day 1 adult (D1) stage worms (**Supplementary Fig. 1a**). The second device, the vivoChip-24x-4L device, has an additional layer (4 layers in total) to reduce the microchannel dimensions further to enable immobilization of smaller larvae state (L4) worms as well (**Supplementary Fig. 1b**). We used the 4-layer (4L) microfluidic chip (vivoChip-24x-4L) for testing toxicants in a wide range of concentrations that may result in widely different sizes of *C. elegans* from young L4 up to D1 adult stage.

#### Pre-processing of images

Images are automatically uploaded to a local server for processing and analysis. Each channel is then cropped into individual hyperstacks by clipping the full FOV hyperstack into eight 150 μm wide sections (Fig. 1e). The cropping is centered around each predicted channel centerline, which is determined in relation to the fiduciary marker (Fig. 1g).

#### Manual segmentation for ground truth data

Manually annotating each volumetric image of individual C *elegans* is done with an in-house graphical user interface (GUI) toolbox (vivoSegmenter). The GUI allows the user to scroll over multiple z-plane images and time points for each cropped channel. Since the morphology of the segmented worm body in a given channel does not change substantially between time points, users only consider the first time point. In the GUI, the user first selects one of the 3 classes for each channel: no worm (empty channel), partial worm (a partially visible worm is present inside the channel), or full worm (a full worm body is present in the channel). Users then segment full worms by clicking multiple points along the worm bodies in different z-slices in each cropped channel presented by the GUI. Specifically, the GUI registers the coordinates (x, y, and z) of the points as the users click on the image. Once all the points are entered and closed to encompass the worm, a polygon is created by connecting all the points as vertices. Most of the *C elegans* body segmentation requires users to examine 3 to 5 z-slices. While depth information is important for clearly delineating boundaries, wide-field microscopy lacks the optical sectioning required to produce fine-grained segmentation over depth. We, therefore, collapse all segmentation polygons, namely our ground truth, into a single 2D binary image. Finally, each channel is assigned a class label (full, partial, or empty). A single polygon is then generated for the full worm class label only.

#### Data pre-processing for ML analysis

Our network is designed to work with either a full z-stack image volume or a subset of the data volume. Specifically, while we collect 10 focal planes per time point during imaging, we have found that successfully solving most vision-related problems was optimally done with a subset of a single volume centered on a relevant focal plane (Fig. 1f). Finding this central focal plane of a cropped channel can be done by taking the Laplace transform of each z-slice and selecting the one with the highest frequency components [39, 40]. We note that the mode (most occurring value) of the z-values from all the vertices the user clicks for worm segmentation is the same z-plane that we estimate as the best focal plane through the Laplace transform. After identifying the central plane, *N* planes from focal planes from each side are collected and stacked along the channel dimension (*H* × *W)* to form a 2.5D tensor, together represented as a (2*N* + 1) × *H* × *W* tensor. For each cropped channel, the height (H) of the image is fixed to 5,056 pixels and the width (W) to some value between 340 ≤ W ≤ 360 pixels depending on the cropping process. To obtain a similar size for all 960 channels and make the size suitable for our network, images are padded on both sides to obtain a fixed width of 384 pixels. In practice, we found using *N* = 1 (3 z-stack images) to be the optimal configuration for this problem, as expanding beyond 3 z-slices did not improve performance.

#### vivoBodySeg architecture for C. elegans body analysis

**Figure 2:**
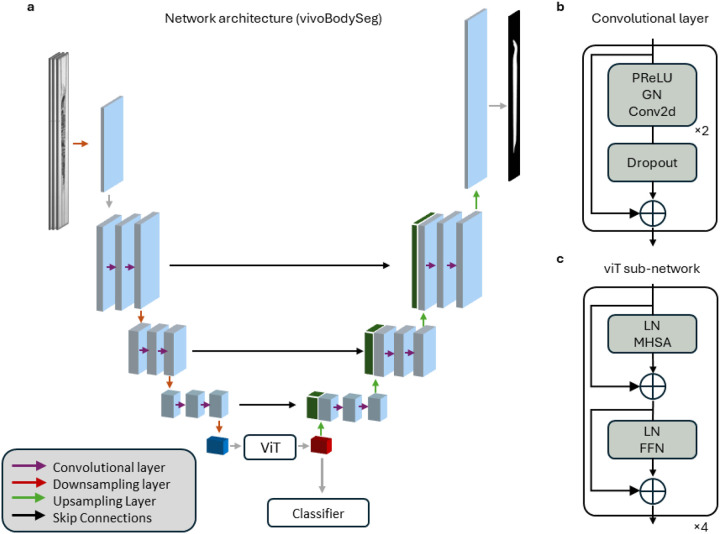
The U-Net architecture of vivoBodySeg for automated analysis of *C. elegans* body. (**a**) Schematic of our 4-layer U-Net. (**b**) Overview of our basic convolutional layer that defines our computation in our encoder and decoder. (**c**) Overview of vision transformer (viT).

We propose a 2.5D U-Net for vivoBodySeg model with an attention mechanism at the bottleneck for the classification and semantic segmentation of *C. elegans* [41]. The proposed architecture consists of the following sub-networks: a fully convolutional encoder, a bottleneck layer consisting of a small vision transformer (ViT), and a fully convolutional decoder (Fig. 2a). This network produces two outputs: a pixel-wise segmentation over classes produced by the decoder and an image-wise classification over classes produced at the bottleneck layer.

Drawing terminology from previous work, an N.5D CNN refers to a convolution neural network (CNN) that processes N + 1 dimensions but only N of them in a convolutional manner [42]. The last dimension is stacked over the channel/feature dimension in a manner similar to how spectral information is often treated. Each layer of our vivoBodySeg network is defined by the same residual convolutional layer in both the encoder and decoder; the general design of this layer follows what is proposed by He *et. al*. in their work on the ResNet architecture (Fig. 2b) [43]. Following the standard practice for models that perform semantic segmentation, the final decoder layer is followed by a set of linear layers and the softmax function to produce a soft segmentation.

To augment the standard convolutional approach, we introduce a ViT at the bottleneck to enable efficient, long-range communication between embedded voxels (Fig. 2c). While standard images collected by non-scientific cameras may span hundreds of pixels, our images with Iris15 camera span over 5,056 pixels along the worm length. By replacing the standard convolutional bottleneck with a ViT, our goal is to ease the classification task as relevant image patches span thousands of pixels. The output of our ViT is routed to two separate sub-networks: the convolutional decoder and the classification subnetwork. The classifier we designed involves the pooled attention mechanism introduced by Lee *et. al*. with a single seed vector followed by a series of linear layers to produce a vector with elements such that we can use it for our classification task [44].

Unlike a standard ViT that generates a tokenized version of our image through a linear embedding of voxels [45] or a secondary generative model such as a VQ-VAE [46], the encoder of our U-Net serves as our embedding mechanism. Upon reaching the bottleneck, the 4D tensor is rearranged into a tokenized format, $X_{e}=\text{Flatten}\left(F_{\text{enc}}\left(X\right)\right)\in {\mathbb{R}}^{B,\frac{H}{S}\times \frac{W}{S},C}$ where *s* is equal to *2*^*Layers*^ and *c* = 256, and combined with learnable positional encodings. Following this step, the data is processed as a sequence by a small 4-layer ViT. Following the standard design introduced by Vaswani *et. al*. for natural language processing, data is first normalized and routed to a multi-head self-attention block. Following a residual connection, data is once again normalized and routed to a feed-forward network where we maintain the standard feature expansion e.g., ***|C***_***FFN***_***|* =** 4 * *|C*_***MHSA***_***|*** [47]. The input and output of the self-attention block are also connected by a residual connection. Our code and network configuration files for the vivoBodySeg framework are available for academic use upon request.

#### Network training, validation, and testing for C. elegans images

The training set includes 3,637 channels acquired using the vivoChip-24x-3L devices from experiments conducted for different treatment conditions. In 81% of the channels, the entire body of *C. elegans* is fully present within the channel (full worm), 14% of these examples are worms only partially visible within a channel (partial worm), and 5% are examples of purely empty channels (no worm). We split the entire data in an 8:1:1 ratio between the training, validation, and testing sets, where the percentages of class distribution are consistent across all sets.

We utilize horizontal and vertical flips, small rotations, and contrast adjustments to enhance the underlying dataset. Each training step randomly selects a mini batch of 32 images and serves as examples in a single forward pass. We use an AdamW optimizer with an initial learning rate of 2×10^−4^ and weight decay of 1×10^−2^ [48]. During training, the learning rate is scheduled according to cosine annealing with warm restarts (*W*_0_ *=* 10, *F* = 2) over 1,200 total epochs [49]. We update our network according to our loss functions for segmentation and image classification. Only full worms are sent to the decoder for learning segmentation, and the weights are updated. All vivoBodySeg networks are trained on a computer with 128 GB of memory and an A6000 GPU with 48 GB of VRAM.

#### Post-processing and inference to find C. elegans body parameters

During inference or testing, we only consider those channels with full worms and apply a simple set of post-processing procedures to clean the data and extract relevant endpoints. During post-processing, a threshold of 0.50 is applied to the output such that we form a binary mask indicative of the *C. elegans* body. After this step, the connected component analysis identifies large binary objects as the worm bodies (trained to detect L4 up to adult stage worms) and removes all small objects outside this binary mask (such as laid eggs, small larvae, debris, etc.) present within the channel. The binary mask is used to estimate three body parameters: length, area, and volume. The body length is retrieved from the longest spanning tree in the skeleton of the binary mask. The binary object provides the area of the *C. elegans* body. The total volume is estimated by taking into account the known height of each pixel inside the predicted *C. elegans* mask.

#### Evaluation metrics for model performance

When evaluating our network, we report several metrics to quantify the overall segmentation quality. To understand general network performance, we use the Dice score to track validation progress and to quantify how the model works in a test setting. We use the Wilcoxon signed rank sum test to assess whether the mean Dice score ranks differ between models. We then use our post-processed data to further elucidate the model accuracy by reporting the ratio of the predicted skeleton length over the ground truth skeleton length. We also estimate the volume ratio (predicted volume to the ground truth volume) and classification accuracy using the weighted F1 score. To compare the model performance with human scorers, we calculate the Dice score from the segmentation for multiple scientists and compare it with the Dice score estimated between the predicted mask and the ground truth segmentation data.

#### Autofluorescence analysis

We measure the autofluorescence signal within the predicted body mask using the fluorescence image captured with a GFP filter set. First, we create a maximum-intensity projection image from all 10 z-stacks. Using the control wells with 0.2% DMSO treatment, we determine a threshold intensity above which the brightest 5% of the pixels lie. These 5% pixels correspond to the granules of lysosomes in the worm gut, which are major contributors to the increase in autofluorescence under stressors. We calculate the average pixel intensity considering the pixels with an intensity above this threshold and within the predicted body mask for all worms. We use the average autofluorescence value per unit body length and per unit body area to identify dose-dependent responses to a chemical treatment.

#### Statistical analysis of dose-dependent body parameters

For developmental toxicity assays, body parameters and autofluorescence signals are calculated for each worm. The individual worm values are filtered to remove all measurements from worms with body lengths and autofluorescence signals significantly deviating from the median using the Tukey fences (1.5 * the interquartile range). Worms with measurements outside these fences are removed from the analysis. After filtering, we use the remaining worms to estimate the well average (μ) and standard deviation (σ) for body length, area, and volume. The data is presented as average ± standard error of the mean (SEM) from multiple replicates. We calculate the coefficient of variance (*CV* = *σ /μ*) from all the control wells. The average values for each body parameter and autofluorescence signal are plotted for different concentrations, and the effective concentration (EC_10_) value for the 10% change in the parameter is fitted from datasets with a 4-parameter, variable slope Hill function using the “Find ECanything” nonlinear fit function of GraphPad Prism. The slope bottom and top are constrained to zero (for length, area, and volume) and left unconstrained (for autofluorescence signal), respectively. The EC_10_ values are presented with ± 95% confidence interval (CI) values for each parameter. To calculate the lowest observable adverse effect level (LOAEL) for each phenotype, we test for normality (Shapiro-Wilk test) and identify the first dose where the phenotype departs significantly (*p*-value < 0.05) from the baseline value of the control population using Welch ANOVA with *post hoc* Dunnett’s T3 multiple comparison tests.

## Results

### A 2.5D U-Net vivoBodySeg model segments C. elegans body.

Making use of our training corpus, we trained a base model (U-Net with no attention, vivoBodySeg-2D) alongside an improved U-Net model making use of the attention bottleneck (vivoBodySeg-2D, Att) and the 2.5D U-Net with an attention bottleneck (vivoBodySeg-2.5D, Att). All three models were trained and validated using our in-house computational power (**Supplementary Fig. 2**). We tested all three models on an unseen *M* = 362 test samples. We calculated segmentation accuracy using the Dice score, length ratio, volume ratio, and classification accuracy for all three models (Table 1). We note the length and volume ratios are highly correlated with predicted body segmentation. We found vivoBodySeg-2.5D, Att to be highly performant, with an average Dice score of 97.80 ± 0.08, a length ratio of 0.991 ± 0.001, and a volume ratio of 1.008 ± 0.002. The vivoBodySeg-2.5D, Att model classified all 362 test images into three groups (301 full worms, 48 partial worms, and 13 no worms) with a weighted F1 score of 0.995. The model could detect *C. elegans* bodies with high accuracy completely inside the field of view and ignore foreign particles with high confidence (**Supplementary Fig. 3**).

**Figure 3:**
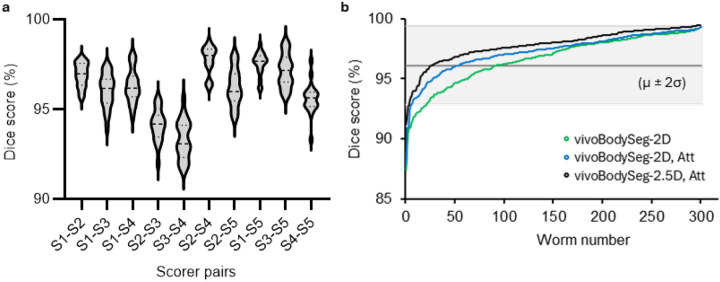
Comparison of Dice scores for body segmentation by multiple scorers and by different vivoBodySeg models. (**a**) Dice scores for each possible pair of five individual scorers for a subset of 20 channels. (**b**) The Dice score for all 301 test samples for three vivoBodySeg models arranged with worm numbers representing low to high Dice scores. The grey area represents the mean ± 2×standard deviation (μ ± 2σ) values for the Dice score from the segmentations of 5 individual scorers. Using the Wilcoxon signed rank sum test, the mean Dice score of vivoBodySeg-2.5D, Att was significantly higher than that of vivoBodySeg-2D and vivoBodySeg −2D, Att (*p*-value <0.001).

While understanding how our model performs with respect to manual segmentations, we wanted to compare our models’ results with respect to a population of human scorers. Five scientists segmented the same 20 channels with *C. elegans* to calculate the values of Dice scores associated with inter-individual variability. We calculated Dice scores between all 5 scorers and found the average to be 96.10 ± 0.12 (n = 200) (Fig. 3a). We then estimated the Dice scores for all three models using 301 body segmentations (under the full worm classification) from two of the scorers as the test sample set. vivoBodySeg-2.5D, Att had 9% of the samples with a Dice score below the average value of 96.10% compared to the 31% for vivoBodySeg-2D (Fig. 3b and **Supplementary Fig. 4a-b**).

#### Few-shot learning to improve the body detection for smaller worms immobilized in vivoChip-24x-4L device

New chemicals are often tested with a wide range of concentrations to identify lethal doses in a dose-finding experiment. In such assays, several worm populations are developmentally arrested or severely retarded, causing small-size worms. In addition, such highly potent chemical conditions cause a heterogeneous worm population with variable body sizes. To study such conditions, we treated *C. elegans* populations with high doses of a reference toxicant CH_3_Hg, where the *C. elegans* are expected to have slow development and thus significantly smaller body sizes and lower image contrasts.

**Figure 4:**
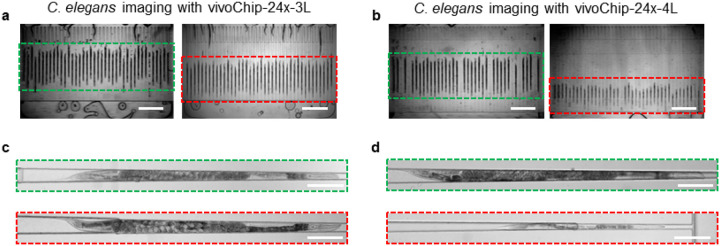
Images of *C. elegans* with developmental defects captured using two vivoChip-24x device designs. (**a**) Image of 40 trapping channels from the 3-layer chip (vivoChip-24x-3L) with immobilized worms from a control population boxed in green (left panel) and a population that was treated with 4 μM CH_3_Hg in 0.2% DMSO boxed in red (right panel). (**b**) Image of 40 trapping channels from a 4-layer chip (vivoChip-24x-4L) with immobilized worms from a control population boxed in green (left panel) and a population that was treated with 8 μM CH_3_Hg in 0.2% DMSO (right panel). Scale bar = 1 mm. (**c, d**) High-magnification images of a single control worm (green box) and a CH_3_Hg-treated worm (red box). Scale bar = 200 μm.

Beyond the mildly dissimilar microfluidic environment, the differences between the larvae and adult *C. elegans* are visually profound. The developmentally retarded worms (young L4) are transparent, have no eggs, and have fewer gut granules than normally grown adult *C elegans* (Figs. 4a–d). Using vivoBodySeg-2.5D, Att, we measured a zero-shot Dice score (94.90 ± 0.47) on the images obtained with vivoChip-24x-4L device with phenotypically different worms (Table 2). Since vivoBodySeg-2.5D, Att was highly performant on images acquired with vivoChip-24x-3L devices (Dice score of 97.80 ± 0.08), we wanted to understand few-shot learning (FSL) performance on a phenotypically different population of worm images that are obtained with vivoChip-24x-4L devices. For this test, we used 512 and 107 channel images from vivoChip-24x-4L for training and testing, respectively. We fine-tuned the network in five steps with varying amounts of training data to understand the impact of dataset size on the fine-tuning process. The training was performed with 32 (6.3%), 64 (12.5%), 128 (25.0%), 256 (50.0%), and 512 (100.0%) training samples from the vivoChip-24x-4L device. To avoid catastrophic forgetting, an equal number of worms from the previous 3L dataset was mixed with this new 4L data. Each split was trained with a batch size of 32 over 250 epochs, where we followed a cosine annealed one-cycle learning rate scheduler and a maximum learning rate of 10^−6^. All the trained models were tested with *M* = 362 (from vivoChip-24x-3L) images and *M* = 107 (vivoChip-24x-4L) images. To understand model improvement, we report the Dice scores and classification accuracies using weighted F1 scores for worm images acquired with vivoChip-24x-3L (adult worms only) and vivoChip-24x-4L (L4 up to adult worms) devices (Table 2 and **Supplementary Figs. 5a-b**).

**Figure 5:**
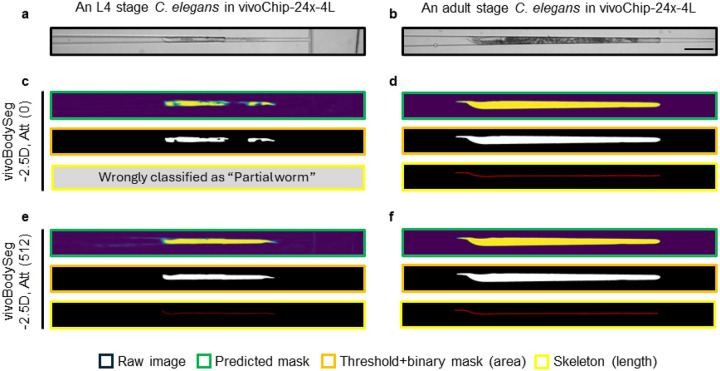
Improved worm detection using the few-shot learning of vivoBodySeg model. Images of a young L4 (**a**) and an adult stage (**b**) *C. elegans,* imaged in the vivoChip-24x-4L device. (**c, d**) Results from the baseline vivoBodySeg-2.5D, Att model with zero-shot learning. The model wrongly classified the channel as having a partial worm and thus discarded it for further analysis. (**e, f**) Results from the vivoBodySeg-2.5D, Att model with few-shot learning using 512 images. The model correctly predicted the worm mask and classified the channel images. Raw image, predicted mask, binary mask, and skeleton are compared. The scale bar is 200 μm.

While phenotypic differences between the two populations reduced the performance of our baseline model (vivoBodySeg-2.5D, Att), we were able to finetune its performance in detecting worm bodies across a wide range of worm populations within four hours of additional training (Figs. 5a–f). While the ideal corpus represents any unseen test set, the fine-tuning strategies deployed here allowed us to process new and highly disparate data classes with hundreds rather than thousands of examples. Although the final vivoBodySeg-2.5D, Att (trained with 512 images) model detected the adults with slightly lower accuracy (Dice score 97.39 ± 0.11 from 97.80 ± 0.08) for images from vivoChip-24x-3L devices, the performance improved immensely for the new class of data (Dice score 96.91 ± 0.19 from 94.90 ± 0.47). The final vivoBodySeg-2.5D, Att (100.0%) model detected adult worms imaged in the vivoChip-24x-3L with similar accuracy as the baseline model, before finetuning (**Supplementary Fig. 6**). We achieved a weighted F1 score of 0.986 using the final vivoBodySeg-2.5D, Att model compared to 0.860 estimated with the baseline model before the FSL approach was implemented.

#### Studying developmental toxicology in C. elegans with methyl mercury exposure

**Figure 6:**
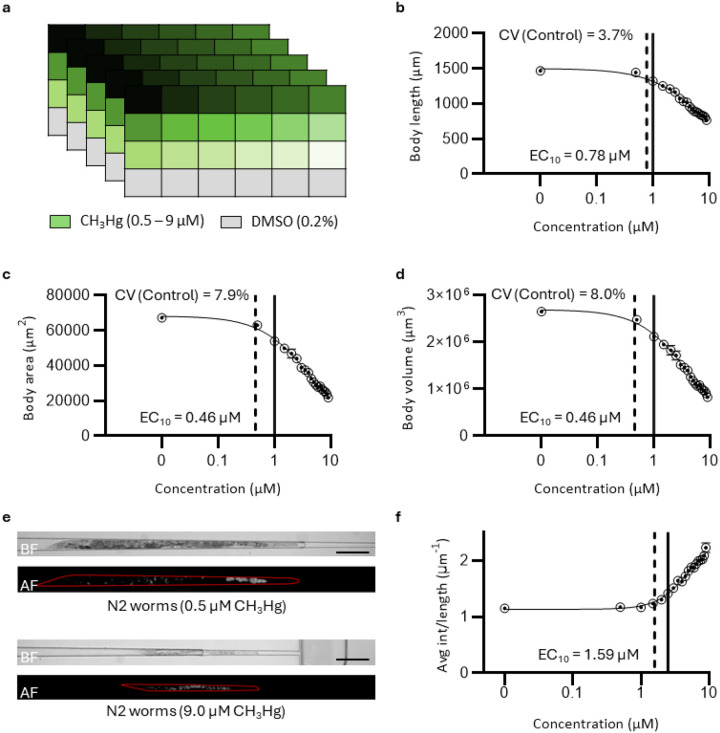
Automated body parameter and autofluorescence analysis using the vivoBodySeg model. (**a**) Map showing exposure of 24 *C. elegans* populations with CH_3_Hg (0.5 – 9.0 μM) and 0.2% DMSO. The experiment was repeated with 5 vivoChip-24–4L devices. The data from all 5 experiments were used to quantify average body length (**b**), body area (**c**), and body volume (**d**). The data are presented as mean ± SEM (*n* = 5 repeats). The solid line represents the Hill-function fit to the experimental data. The dotted line represents the EC_10_ for each parameter. The coefficient of variation (CV) is indicated for the control wells. (**e**) Example images of worms from 0.5 μM and 9.0 μM CH_3_Hg. Brightfield (BF) and autofluorescence (AF) images obtained using the GFP filter are shown. The scale bar is 200 μm. (**f**) Average autofluorescence intensity per unit body length for CH_3_Hg and DMSO control populations. The data are presented as mean ± SEM (*n* ≥ 4 repeats). The dotted line represents the EC_10_ value. The solid line represents the LOAEL value of 1.0 μM for length, area, and volume (**b – d**) and 2.5 μM for Avg int/length (f).

We exposed age-synchronized *C. elegans* larvae to 12 concentrations of CH_3_Hg in 0.2% DMSO for 72 hours in plastic well plates and imaged them using the vivoChip-24x-4L device. The experiment was repeated five times to identify batch-to-batch variability and estimate the coefficient of variability parameters from the 0.2% DMSO controls (Fig. 6a). We analyzed all the 4,800 microfluidic channels from 5 independent experiments using the final vivoBodySeg model (few-shot learning with 512 images) to automatically identify channels with *C. elegans* and estimate the body parameters (length, area, and volume). The inference code with this model analyzed a full chip with 1,200 brightfield images in 35 minutes. We obtained highly similar assay results in all the body parameters, studied from all five chip experiments. The coefficient of variance (CV) between the DMSO controls from 5 replicates for the body length (3.7%), body area (7.9%), and body volume (8.0%) were much below the 30% threshold, accepted CV values in a similar guideline using a similar species in the OECD test guideline [50]. The values indicate that we have a robust automated developmental toxicity (DevTox) assay using *C. elegans* models that can detect small changes in the body parameters with high confidence.

To demonstrate the utility of our DevTox assay, we performed a dose-response study on the CH_3_Hg, a reference toxicant tested in *C. elegans* and other species including humans [17, 37, 38, 51–55]. The average body parameters from five experiments consistently decreased with increased CH_3_Hg concentration (Figs. 6b–d). The body volume (EC_10_ = 0.46, 0.32–0.63 μM, ± 95% CI) and body area (EC_10_ = 0.46, 0.32–0.64 μM) show toxicity effects at slightly lower concentrations than the body length (EC_10_ = 0.78, 0.56–1.05 μM). The LOAEL values for all three body parameters were estimated as 1.0 μM. The worms exposed to the highest dose (9.0 μM) of the toxicant only develop to the early L4 stage. These smallest worms were, thus, trapped in the fourth layer of the microchannels, towards the exit with the smallest channel height, matching our body parameters calculated using the model (Fig. 6e).

The autofluorescence signal in *C. elegans,* as in mammalian cells, is found in the granules of lysosomes, which are present in their intestine [56]. The intestinal autofluorescence is known to increase with age of the worm [57–59] and when exposed to toxicants [60, 61]. We utilized predicted body masks to analyze the autofluorescence signal within each *C. elegans* body and identify the change in the average autofluorescence signal in a quantitative manner. The average autofluorescence signal per unit body length for 9.0 μM CH_3_Hg (2.23 ± 0.09) is ~ 2x higher than the value for 0.5 μM CH_3_Hg (1.17± 0.06) in a statistically significant manner (*p*-value < 0.001). The average autofluorescence signal per unit body length for 0.5 μM CH_3_Hg is similar to the baseline value for DMSO control (1.15± 0.01, *p*-value = 0.63, Fig. 6f). On the other hand, the average autofluorescence signal per unit body area for worms treated with 9.0 μM CH_3_Hg (0.081 ± 0.008) increased by ~ 3x than the value for 0.5 μM CH_3_Hg (0.027 ± 0.001, *p*-value = 0.002, **Supplementary Fig. 7**). The EC_10_ values for the average autofluorescence signal per unit body length (1.59 μM, 95% CI is 1.27–1.93 μM) is lower than the value for per unit body area (2.98 μM, 95% CI is 2.48–8.44 μM). The LOAEL values for autofluorescence per unit length and per unit area are 2.5 μM and 1.5 μM, respectively. The increase in the autofluorescence signal was delayed compared to the developmental features and is likely to be a less sensitive parameter for toxicology assessment for CH_3_Hg. Additionally, we found that *C. elegans* exposed to high doses of CH_3_Hg showed slow motility when observed in their culture plates, agreeing with the previously published correlation between high autofluorescence and locomotion [61].

## Discussion

This study presents an ML-based image analysis platform to perform DevTox studies using *C. elegans* as a NAMs model. This platform enables rapid high-content analysis of thousands of *C. elegans* images collected as they are immobilized in parallel channels of a large-scale microfluidic platform, vivoChip-24x. DevTox assessment is one of the mandatory tests needed for human and environmental risk assessment of new chemicals before they are approved for commercial use. Due to the large number of chemicals currently in commercial use (85,000) and more than 2,500 new ones added every year [62], industries are seeking innovative solutions to assess chemicals in a high-throughput manner with high predictive power. *C. elegans* has been used as one of the *in vivo* models for early drug discovery and toxicology screens. To improve assay sensitivity, capture multiple phenotypes, and facilitate high-content image-based analyses, we developed an automated imaging and image-analysis pipeline to quantify multiple phenotypes in *C. elegans* models. We used vivoChip technology to capture brightfield, fluorescence, multiple z-stack, and time-lapse images from 960 *C. elegans* and 24 populations in each vivoChip device. Our vivoChips are fabricated using plastic instead of PDMS to lower chemical absorption. The microfluidic channels are sealed with a thin substrate to allow the capturing of high-resolution images with sufficient contrast from transparent worm body parts such as the tail tip or young larvae with reduced gut granules.

The ML-based vivoBodySeg, trained with the images obtained using vivoChip-24x devices, is highly performant in two different experimental settings using 3-layer and 4-layer devices. By including a ViT at the bottleneck layer of our encoder, we can easily communicate features that span the entire image length in a memory-efficient manner. Further, by accounting for the information from multiple z-stack images in a multiplicative manner, the overall network generates volumetric features without relying on higher dimensional convolutions. We find this model highly accurate and indistinguishable from our highly trained scientists for segmenting an arbitrary dataset. The model can analyze data from one vivoChip-24x experiment (~ 1,000 animals, 9,600 single-channel images, 36 GB data) in 35 minutes using a single desktop PC (with 128 GB of memory and an A6000 GPU with 48 GB of VRAM) compared to 5,000 minutes for uninterrupted expert analysis hours.

Our *C. elegans* DevTox analysis includes accurate measurements of worm length, body area, and volume, as they prove to be highly useful developmental endpoints with different levels of sensitivities. Automated analysis of *C. elegans* developmental parameters is currently available using length estimation with the COPAS Biosorter via 1D signal analysis or plate readers via low-resolution image analysis [21–23, 27]. While several groups have used these tools to analyze *C. elegans* bodies, these assays provide data with a high amount of variability and poor statistical power [26]. Advancements in microfluidic technologies have enabled on-chip *C. elegans* imaging and quantifying body parameters using conventional image processing [63, 64]. Unfortunately, these platforms acquire low-resolution images and introduce a high amount of variability in their body parameters. The machine learning pipeline that we developed can analyze the data obtained from our fully automated and scalable vivoChip-24x devices and provide body measurements with high statistical power. Based on the average observed variance in the body parameters, we found a > 80% power to identify a 4% change in length and area, and a 7% change in volume using experimental replicates. The fully automated ML-based approach eliminates user bias, does not suffer from user fatigue, helps reduce assay costs, and achieves high throughput to screen many chemicals in a low-resource setting.

To demonstrate the implementation of our DevTox assay powered with ML analysis, we conducted a study with a well-characterized toxicant CH_3_Hg at 18 different doses ranging from 0.5 to 9.0 concentrations and 0.2% DMSO solvent. We quantified DevTox using the effects on the body length, area, and volume parameters in a dose-dependent manner. We found lower EC_10_ for the body area and volume (0.46 μM) than the body length (0.78 μM) in the N2 strain, indicating the area and volume parameters are more sensitive parameters of DevTox. We noticed that the worm populations with severe developmental defects due to exposure to higher concentrations of CH_3_Hg have higher intestinal autofluorescence signals than the control worms, indicating a possible increase in stress levels from toxicity response. During autofluorescence analysis, we found a few examples where the predicted body mask, especially the tip of the tail or the head of a worm, did not match completely due to body movement between frames. This problem is less concerning for autofluorescence analysis due to the signal being localized to the body region that is completely immobilized. To improve immobilization and hold the entire worm body still during the imaging, the worms can be immobilized with a low concentration of anesthetic solution or gel material.

The machine learning pipeline, combined with our scalable microfluidic technologies, can provide rapid DevTox parameters of new and in-use active ingredients using *C. elegans* models. To the best of our knowledge, this is the first study demonstrating automated, high-throughput quantifications of *C. elegans* volume, which is analogous to body weight measurements as used in standard DevTox and ecotoxicology tests with animals. In the future, we aim to expand the DevTox assays with other endpoints such as reproduction endpoints using *in-utero* embryonic phenotypes. The DevTox parameters have high statistical power and can provide toxicology endpoints from *C. elegans* models for read-across strategies, which are currently being developed by several industries to establish NAMs and understand fit-for-purpose toxicology assays.

## Figures and Tables

**Table 1:** Test results of the proposed U-Net models using 362 images

Model name	Dice score	Length ratio	Volume ratio	Weighted F1 score
vivoBodySeg-2D (no attention)	96.62 ± 0.12	0.987 ± 0.002	1.026 ± 0.003	0.986
vivoBodySeg-2D, Att (with attention)	97.24 ± 0.10	0.988 ± 0.001	0.999 ± 0.003	1.000
vivoBodySeg-2.5D, Att (2.5D, with attention)	97.80 ± 0.08	0.991 ± 0.001	1.008 ± 0.002	0.995

**Table 2:** Dice score for the few-shot learning of vivoBodySeg-2.5D, Att model with different amounts of training data (6.3% – 100% of 512 images).

Number of images (%)	vivoChip-24x-3L device		vivoChip-24x-4L device	
	Dice score	Weighted F1 score	Dice score	Weighted F1 score
0 (0.0%)	97.80 ± 0.08	0.995	94.90 ± 0.47	0.860
32 (6.3%)	96.57 ± 0.14	0.938	95.90 ± 0.31	0.986
64 (12.5%)	96.95 ± 0.12	0.971	95.93 ± 0.30	0.986
128 (25.0%)	97.28 ± 0.11	0.992	96.52 ± 0.32	0.986
256 (50.0%)	97.32 ± 0.10	0.995	96.90 ± 0.25	0.986
512 (100.0%)	97.39 ± 0.11	0.995	96.91 ± 0.19	0.986

## Data Availability

The datasets generated during and/or analyzed during the current study are available from the corresponding authors upon reasonable request.
